# Restoratively driven planning for implants in the posterior maxilla - Part 1: alveolar bone healing, bone assessment and clinical classifications

**DOI:** 10.1038/s41415-023-6391-7

**Published:** 2023-10-27

**Authors:** Elizabeth M. King, Jonathon Schofield

**Affiliations:** 41415444179001 Consultant Senior Lecturer in Restorative Dentistry, University of Bristol, Bristol Dental School, UK; 41415444179002 Senior Clinical Lecturer, University of Bristol, Bristol Dental School, UK

## Abstract

Sinus augmentation in conjunction with implant placement is widely considered to be a predictable and successful treatment option for the edentulous posterior maxilla. However, the anatomical changes of the posterior maxilla following tooth extraction (namely alveolar atrophy and pneumatisation of the maxillary sinus) creates unique challenges for implant and prosthodontic rehabilitation. A large volume of literature has been published regarding the surgical indications and treatment planning for implants in the posterior maxilla. In comparison, there is a relative paucity of literature regarding the prosthodontic challenges associated with implants placed in augmented maxillary sinuses. This article describes the scientific background of native and grafted alveolar bone healing in relation to implant rehabilitation. Furthermore, clinical classifications available to assist implant treatment planning are described.

**Supplementary Information:**

Zusatzmaterial online: Zu diesem Beitrag sind unter 10.1038/s41415-023-6391-7 für autorisierte Leser zusätzliche Dateien abrufbar.

## Key points

- Alveolar bone changes following tooth extraction complicate implant planning, placement and restoration in the posterior maxilla.
- A large volume of literature has been published regarding the surgical planning for implants in the posterior maxilla despite the importance of restoratively driven planning.
- Numerous classifications can be applied to help plan implant treatment in the posterior maxilla but none have the details required for restoratively driven planning.

**Supplementary Information:**

Zusatzmaterial online: Zu diesem Beitrag sind unter 10.1038/s41415-023-6391-7 für autorisierte Leser zusätzliche Dateien abrufbar.

## Introduction

With the increasing prevalence of dental implants for the restoration of missing teeth, bone grafting procedures have also increased in prevalence to enable implants to be placed in more challenging sites. The anatomy of the posterior maxilla changes significantly following tooth extraction due to alveolar ridge resorption and maxillary sinus pneumatisation, resulting in reduced alveolar bone width and height. Of the numerous bone grafting techniques used to increase vertical bone height, sinus augmentation provides the most predictable implant survival rates with the reduced need for a donor site, and thus is the most common bone grafting procedure used in the posterior maxilla.^1^ Sinus augmentation most commonly involves accessing the maxillary sinus (either through the lateral wall or by a transalveolar route), lifting the Schneiderian membrane and placing either autogenous or xenogenous bone. Implant placement is either performed simultaneously (at the time of bone grafting) or after a period of graft consolidation (usually six or more months), depending on the residual native maxillary bone height.

To achieve the best functional and aesthetic implant treatment outcomes, prosthodontic planning should drive surgical planning. However, as implant planning in the posterior maxilla often requires a considerable degree of surgical planning, the prosthodontic aspects of treatment planning can easily be overlooked. This inclination is reflected in the dental literature, with the majority of articles and classifications focusing on the surgical aspects of planning. In comparison, there is a relative paucity of literature describing the prosthodontic challenges associated with implants placed in the posterior maxilla and/or augmented maxillary sinuses.

**Fig. 1:**
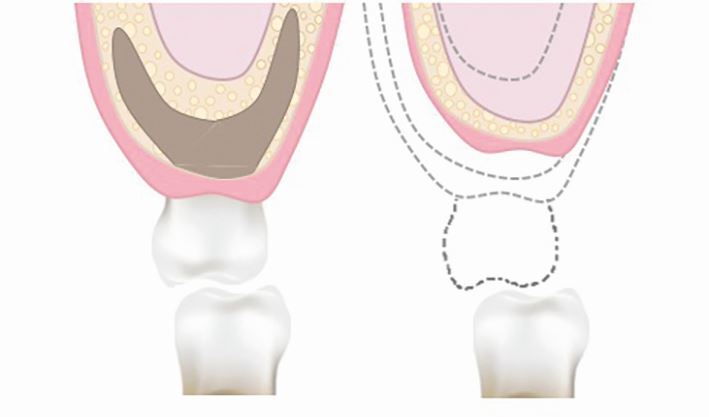
Diagram showing the anatomical changes in the alveolar ridge as a result of bone resorption and sinus pneumatisation following tooth extraction

It is clear from clinical evidence that implants in the posterior maxilla have the lowest survival rate compared to other regions in the mouth.^2^^,^^3^ Furthermore, implants placed in augmented maxillary sinuses have shown to have a lower survival rates than those placed in native posterior maxillary bone.^4^^,^^5^ The bone of the posterior maxilla has fine trabeculae and thin buccal cortical plates which provides poor bone quality for the placement of dental implants.^6^ When posterior maxillary teeth are extracted, the alveolar ridge undergoes vertical and horizontal resorption (Fig. 1). In addition, the floor of the maxillary sinus can pneumatise and expand in an inferior direction (Fig. 1). This results in a clinical situation where the residual vertical height and width of the alveolar ridge is significantly reduced and thus minimises the amount of available bone for dental implant placement.

Masticatory forces in the posterior maxilla are three times greater than the anterior maxilla, with the second molar tooth exerting the greatest occlusal force.^7^ Occlusal loads are short and intermittent, with approximately 15-20 minutes peak loading over a 24-hour period.^8^ Forces may load teeth axially or eccentrically, with the average occlusal force ranging from 50-300 N. However, parafunctional occlusal habits result in a significant increase in duration and frequency of occlusal forces, with forces up to 800 N possible.^9^ Therefore, implant-retained restorations in the posterior maxilla are exposed to significant biomechanical stresses compared with other intra-oral sites. Taking these factors into consideration, it is evident that implant placement in the posterior maxilla can present a prosthodontically and surgically challenging situation.

To aid comprehensive clinical assessment, numerous classifications have been developed to provide systematic protocols for treatment planning dental implants (plus any required bone augmentation procedures). Such classifications encourage objective clinical assessment, facilitate the identification of case complexity and can provide a guide for clinical decision-making. Furthermore, classifications assist clinical record-keeping, inter-professional communication and help standardise reporting in the scientific literature. Having a thorough knowledge of the different classifications related to dental implant planning and potential adjunctive bone grafting is advantageous for clinicians involved in the prosthodontic and surgical treatment of such cases.

The aim of this paper is to describe the alveolar bone changes that affect implant planning and prosthodontic delivery and describe the classifications available to assist the planning for implants in the posterior maxilla.

## Alveolar bone changes in the posterior maxilla

Alveolar bone volume directly influences prosthesis design and implant position. As alveolar bone volume decreases, the complexity of prosthesis and implant delivery increases, with significant bone resorption rendering implant placement and restoration unviable without adjunctive bone augmentation techniques. It is therefore important to understand the alveolar bone changes in the posterior maxilla following tooth extraction.

The horizontal and vertical shape of the posterior alveolar ridge is determined by tooth position. The average width of the dentate posterior maxilla is approximately 9.57 mm in the second premolar region and 12.38 mm in the first molar region.^10^ The average posterior maxillary alveolar bone height in relation to the maxillary sinus is approximately 7.8-8.1 mm in dentate individuals. Following tooth extraction, alveolar bone resorption occurs with approximately 3.87 mm loss in the horizontal dimension and 1.67-2.03 mm loss in the vertical dimension.^11^ Most alveolar resorption occurs within six months of extraction, with 30% occurring within the first 12 weeks.^12^ Additionally, after tooth loss, the maxillary sinus pneumatises inferiorly, which results in a further reduction in alveolar bone height. Post-extraction expansion of the maxillary sinus in an inferior direction is approximately 1.83-2.83 mm, with larger sinus pneumatisation of approximately 2.91-3.56 mm observed in the second molar region.^13^ Post-extraction sinus pneumatisation occurs within 4-6 months following extraction.^13^ Certain local factors increase the likelihood of maxillary sinus pneumatisation and these include: teeth surrounded by a superiorly curving sinus floor, tooth roots shown to protrude into the sinus cavity by computerised tomography (CT) imaging, extraction of second molars, extraction of several adjacent posterior teeth, and extraction of a tooth with missing adjacent teeth.^13^ Identifying these factors pre extraction can help identify patients who may be at risk of sinus pneumatisation in the short- and long-term.

For dentists involved with the placement or restoration of implants, it is important to be able to assess and predict these alveolar bone changes clinically and radiographically as a result of tooth loss. By doing so, the prosthodontic and surgical complexity of a case can be determined pre-operatively. Classification tools can be effective in helping achieve this.

## Assessment of alveolar bone

Thorough assessment of alveolar bone is the foundation of comprehensive prosthodontic, surgical and implant planning, and enables planning of the different treatment stages listed in Table 1. An implant and the accompanying implant-supported prosthesis relies on adequate bone volume and bone strength to provide support during loading. Loading of a prosthesis will occur during mastication, swallowing and parafunction. The loads transmitted to bone induce strain. A strain of 1,000 microstrain is a change in bone length of 0.1% compared to the original bone length. An 'adapted window' of 50-1,500 microstrain gives rise to an equilibrium of bone modelling and remodelling whereby bone levels around an implant are maintained.^14^ However, in the posterior maxilla, the bone volume and strength is often compromised and the potential to exceed the 'adapted window' is high. Prosthodontic and implant planning should take into account the bone volume and strength in this region to reduce the risk of occlusal overload.

**Table 1:** Treatment planning outcomes in relation to alveolar bone assessment

Prosthodontic planning	Position of the dental prosthesis/prosthetic teeth
	Provisional prosthesis design
	Definitive prosthesis design
Surgical planning	Maxillary sinus augmentation Transalveolar technique Lateral wall technique
	Alveolar ridge augmentation
	Vertical
	Horizontal
Implant planning	Position
	Length
	Diameter
	Number
	Timing of implant placement Simultaneous, with sinus augmentation Delayed

### Bone volume

Bone volume can be examined clinically through visual assessment and palpation of the edentulous ridge, or radiographically using measurements from conventional radiographs and cone beam computed tomography (CBCT) scan data. If reduced bone volume is identified, it is important to plan at the outset whether this will be overcome using prosthodontic techniques or surgical techniques (Table 2).

**Table 2:** Prosthetic and surgical techniques to overcome reduced bone volume in the horizontal and vertical dimensions

	Prosthodontic techniques		Surgical techniques
Horizontal	Buccally cantilevered prosthesis	Removable flanged prosthesis	Horizontal ridge augmentation
	Alternative posterior occlusal relationship		
Vertical	Increased crown/prosthesis height		Vertical ridge augmentation
			Sinus augmentation

Numerous bone volume classifications have been published to aid objective assessment of alveolar bone levels. Essentially, bone volume is clinically assessed by examining bone width, bone height and inter-arch relationships. Bone volume assessment begins the process of prosthodontic planning, as it identifies whether there is enough bone to place both the teeth and implants in their ideal position, or whether surgical intervention is necessary to improve the prosthodontic envelope.

Bone width is defined as the distance between the buccal and the palatal/lingual plates, measured at the crest. With regards to surgical planning, a dental implant should be surrounded by a minimum of 1 mm of buccal and palatal/lingual bone. The ideal bone width depends on the diameter of implant being selected, with a conventional implant diameter ranging from 3.75-4 mm.^15^ Therefore, a minimum of 6 mm bucco-lingual/palatal width is required if using a conventional diameter implant. Narrow implants (less than 3.75 mm in diameter) are contraindicated in the posterior maxilla.^16^

Bone height for implant placement is measured from the alveolar crest to proposed depth of implant placement, or to the level of an associated anatomical structure if present. It is recommended that a minimum margin of 2 mm from vital structures is respected; however, implants in the posterior maxilla can be planned to engage the bone of the sinus floor or penetrate the sinus floor if sinus augmentation is performed. Conventional implants have a length of ≥10 mm, so traditionally, 10 mm+ of alveolar bone is recommended for predictable implant placement. Most indices and sinus augmentation techniques developed for implant placement in the posterior maxilla are based on the ability to achieve an implant placement of 10 mm length.

Vertical and horizontal inter-arch relationships can be affected by alveolar resorption. Vertical resorption can lead to increased inter-arch distance and horizontal resorption can result in an unfavourable horizontal relationship of the maxillary and mandibular ridges/teeth (Fig. 1).

### Bone strength

Understanding bone strength in the posterior maxilla is crucial to plan predictable short- and long-term dental implant treatment. Due to the fine trabecular bone structure, bone strength in the posterior maxilla is low compared to the bone in other areas of the maxilla and mandible. In the short-term, reduced bone strength can result in reduced implant primary stability. As achievement and maintenance of implant stability are prerequisites for successful osseointegration, implants with poor primary stability have a higher risk of failure.^17^ In the long-term, reduced bone strength is related to a higher risk of implant failure following prosthetic loading, and occlusal overload has been suggested as a possible risk factor for marginal bone loss and implant loss.^2^^,^^3^^,^^18^

**Fig. 2:**
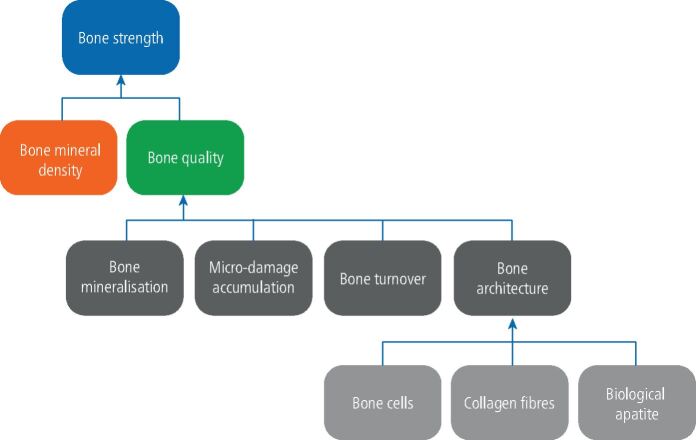
The biomechanical function of bone as a combination of bone quality and bone strength. Reprinted and adapted from *Journal of Prosthodontic Research*, vol 61, Kuroshima *et al*., 'A paradigm shift for bone quality in dentistry: A literature review', pp 353-362, 2017, with permission from Elsevier^19^

Bone strength is determined by the bone mineral density (BMD) and the bone quality (Fig. 2).^19^ Bone quality is independent of BMD and is determined by bone architecture, turnover, damage accumulation (for example, microfractures) and mineralisation.^20^ Furthermore, bone architecture is defined by the number and viability of bone cells, orientation and degree of crosslinking of collagen fibres and the texture and orientation of biological apatite crystals in bone.^19^ The only true method of assessment of bone strength is histological examination. As attainment of bone histology is not clinically feasible, secondary methods to assess the quality of bone can be utilised; namely radiographic assessment using CBCT imaging or intra-operative assessment using tactile feedback.

Accurately assessing bone quality pre-operatively is challenging, if not impossible. Hounsfield units (HUs) from CBCT scans have been suggested to identify alveolar bone quality. HUs, which are used to measure the density of a pixel in a CT image, provide a quantitative assessment of bone quality. Strong correlations have been found between medical-grade CT mean bone density using HUS, implant insertion torque and resonance frequency analysis.^21^ However, HUs were originally used to measure radiodensity on medical-grade CT scans and the use of HUs in CBCT is complicated because of crucial differences in the radiophysics between the two scans. Therefore, although HUs can offer some information about radiographic bone quality, there is a risk of unreliability when HU scales are used to measure alveolar bone quality on CBCT scans.^22^

The most commonly used intra-operative methods to objectively measure primary stability are insertion torque values (ITV), which measures rotational stability and implant stability quotient (ISQ), which measures axial stability. A minimum of 32 Ncm ITV has been shown to be necessary for implants to achieve osseointegration, and an ISQ between 55 and 85 is considered acceptable stability at the time of implant placement.^23^^,^^24^ ITVs and ISQs have been shown to be reduced in the posterior maxilla, with significantly reduced ITVs and ISQs detected for implants placed via sinus augmentation procedures.^25^ Using these methods to estimate bone strength enables the restoring dentist to design a prosthesis with features to minimise occlusal overloading if necessary.

### Implants in grafted sinuses

**Fig. 3:**
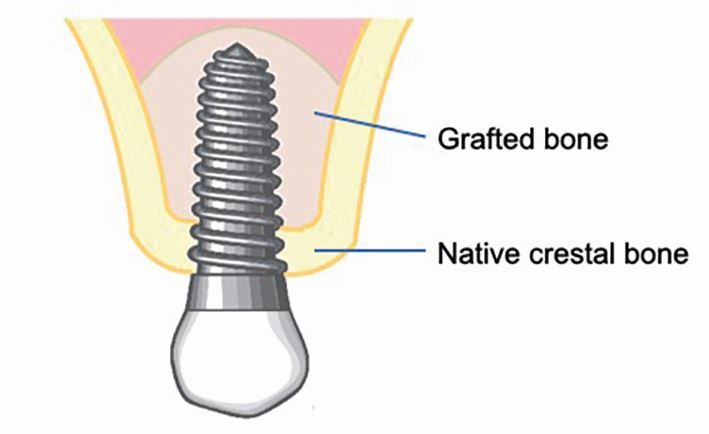
Diagram illustrating the portion of implant in native crestal versus grafted bone in maxillary augmented sinuses when minimal native ridge height is present

Implants in grafted maxillary sinuses are at a higher risk of biomechanical overload due to the physiology of the native and grafted bone surrounding them. The coronal portion of the implant is positioned in native crestal bone and the apical portion is positioned in grafted bone (Fig. 3). It is important to consider the histological maturation of the grafted bone to understand how the forces from occlusion are transmitted to both native crestal and grafted bone.

Bone in grafted sinuses matures over a period of months.^26^ At three months, the graft is considered immature as new bone is not in apposition to xenograft granules which are enveloped by loose connective tissue. By six months, most xenograft particles are surrounded by newly formed immature bone; this is considered an appropriate time for delayed implant placement in larger augmentation cases. The typical histological structure of bone begins to appear at around nine months, and continued bony maturation is detected up to four years and more.^26^ Photoelastic models have demonstrated that implants in immature grafted bone transmit significant stress to the native cortical bone, with a high level of stress transmitting to the grafted bone, with the native bone acting as a 'fulcrum' for stress transfer.^27^^,^^28^ Conversely, when the graft is mature and reaches the same stiffness as native bone, more equitable stress along the implant body is shown. Therefore, implants in augmented maxillary sinuses should be considered at a higher risk of overload, particularly those in more extensive bone grafts and grafts of less than one year. Masticatory forces should be carefully controlled during healing and loading through judicious prosthesis design.

## Implant planning classifications

Numerous classifications are available to aid clinical assessment for the treatment planning of dental implants and adjunctive procedures, such as alveolar bone augmentation. Such classifications can be applied to planning implants in the posterior maxilla, with some being specifically designed for this clinical situation. Table 3 and Table 4 and the online Supplementary Information detail the classifications which can be used to aid implant planning in the posterior maxilla. For the purpose of this article, these classifications have been broadly categorised into those designed for general implant planning (online Supplementary Information), those designed for implant planning in the posterior maxilla (Table 3) and those designed to assess bone quality (Table 4).

**Table 3:** Classifications for implant planning in the posterior maxilla This table contains only relevant elements of each classification. Please see the original publications for the full classification details

Authors	Description	Factors assessed	Classification categories	Case complexity/severity				Treatment recommendations			
Jensen^38^	Radiographic assessment of vertical bone height in posterior maxilla with associated treatment recommendations	Alveolar ridge Implant planning Sinus augmentation	**Class**	**Bone height (mm)**				**Implant timing**		**Sinus augmentation technique**	
			A	10				Immediate possible		Membrane elevation	
			B	7-9				Immediate possible		Transcrestal	
			C	4-6				Immediate or delayed		Lateral window	
			D	0-3				Delayed		Lateral window	
Misch^39^	Radiographic assessment of vertical bone height in posterior maxilla with associated treatment recommendations	Alveolar ridge Implant planning Sinus augmentation	**Subantral (SA) group**	**Bone height (mm)**				**Implant timing**	**Sinus graft technique**		**Healing period**
			SA 1	>12				Conventional	NA		Implant: 4-6 months
			SA 2	10-12				Simultaneous	Transcrestal		Implant: 6-8 months
			SA 3	5-10				Simultaneous/delayed	Lateral window		Graft: 2-4 months Implant: 4-8 months
			SA 4	<5				Delayed (6-10 months)	Lateral window		Graft: 6-10 months Implant: 4-10 months
Juodzbalys and Kubilius^40^	Divides maxilla and mandible into aesthetic and non-aesthetic zones and specifies the risk of implant placement in each. Maxillary sinus region (MSR) is defined. (Only the maxillary non-aesthetic zone from the index is described in this table)	Alveolar ridge Implant planning Ridge augmentation Sinus augmentation Prosthetic planning	**Type**	**Height (mm)**	**Width (mm)**	**Length (mm)**	**Alveolar ridge-vertical position (mm)**	**Treatment recommendations**			
			Type I (low risk)	>10	>6	7-12	≤3	No augmentation			
			Type II (moderate risk)	8-10 (MSR 4-10)	4-6	6-13	3-7	Vertical augmentation +/- sinus lift with simultaneous implant placement			
			Type III (high risk)	<8 (MSR <4)	<4	6-13	>7	Vertical augmentation +/- sinus lift with delayed implant placement

**Table 4:** Classifications assessing bone quality. This table contains only relevant elements of each classification. Please see the original publications for the full classification details

Authors	Description	Classification			Typical location
Leckholm and Zarb^30^	Classifies edentulous maxillae and mandibles according to the degree of resorption	I) Homogenous cortical bone			Anterior mandible
		II) Thick compact bone around a core of dense trabecular bone			Posterior mandible/anterior maxilla
		III) Thin compact bone around a dense trabecular bone. Favourable strength			Posterior maxilla
		IV) Thin cortical bone surrounding a core of low-density trabecular bone			Tuberosity region
Misch (2021)^42^	Classifies edentulous maxillae and mandibles according to the degree of resorption	**Description**	**Tactile analogue**	**Hounsfield units**	
		D1 - dense cortical	Oak/maple	>1,250	Anterior mandible
		D2 - porous cortical and coarse trabecular	White pine/spruce	850-1,250	Anterior and posterior mandible, anterior maxilla
		D3 - porous cortical and fine trabecular	Balsa wood	350-850	Posterior mandible, anterior and posterior maxilla
		D4 - fine trabecular	Styrofoam	150-350	Posterior maxilla
		D5 - osteoid	Soft Styrofoam	Poorly mineralised bone graft	Grafted sites

### General implant planning

Due to their broad scope, classifications for general implant planning can be applied to implant sites anywhere in the maxilla or mandible. All are based on the clinical assessment of alveolar ridge shape and are therefore simple to use to approximate bone volume before implant treatment. The simplest classifications categorise alveolar bone via the description of ridge shape only,^29^^,^^30^^,^^31^^,^^32^ whereas more detailed classifications quantify the severity of alveolar resorption (for example, in millimetres) to provide objective assessment of the alveolar ridge size.^33^^,^^34^^,^^35^ Cawood and Howel and Leckholm and Zarb define case severity by classifying the shape of the alveolar ridge. Although not specific to implant planning, they can be used for the preliminary planning stages for implants or bone augmentation. The Siebert, modified Siebert and horizontal, vertical and combination classification (HVC) define case severity by quantifying the degree of alveolar ridge resorption.^31^^,^^32^^,^^33^^,^^35^ These classifications were specifically designed to assess alveolar bone before ridge augmentation procedures and are useful to approximate whether sufficient alveolar bone is present for implant placement, or whether adjunctive ridge augmentation is required. The HVC classification also provides recommended treatment options (for example, bone augmentation and implant protocols) for different clinical ridge shapes. However, all treatment options recommended are surgically, not prosthodontically, focused. Misch and Judy classify case severity using implant angulation and crown height, as well as alveolar ridge shape.^34^ Including implant angulation and crown height is an important step towards restoratively led planning; however, the recommended treatment options provided within this classification only describe surgical options.

The International Team for Implantology's (ITI) Simple, Advanced, Complex (SAC) tool and Always, Between, Complex (ABC) risk-assessment tool are more comprehensive assessment tools designed for general implant planning.^36^^,^^37^ Among the large range of factors assessed in each classification, both include alveolar bone assessment to define case complexity and can therefore be used to plan implants in the posterior maxilla. The ITI SAC is an online tool developed by the ITI which differentiates between case complexity using numerous surgical and prosthodontic criteria.^36^ By completing a survey-style checklist which guides the user through relevant clinical information, cases are categorised as 'simple', 'advanced' or 'complex'. As part of the ITI SAC tool, the severity of horizontal and vertical alveolar bone defects are categorised by appraising whether bone augmentation is required. Proposed augmentation options are provided for each ridge category, including sinus augmentation for posterior maxillary sites in the 'complex' category. The ABC Risk Score for Implant Treatment is a tool design to aid treatment planning of dental implants using medical history, local factors, surgical factors and restorative factors.^37^ Using this information, cases are categorised as low risk ('always'), medium risk ('between') and increased risk ('complex'). Similarly, the ABC risk score assesses the alveolar ridge in the horizontal and vertical dimensions to establish the overall complexity of a case. Treatment recommendations for bone augmentation, including sinus augmentation, are provided for each ridge category. In both classifications, minimal clinical description is provided to differentiate between the ridge categories but measurements are not provided to quantify ridge defects. Both recommend sinus augmentation for posterior maxillary vertical ridge deficiencies; however, neither provide detail regarding relevant diagnostic criteria (for example, radiographic assessment) of prosthodontic treatment options for such cases. Therefore, these classifications are useful to initiate the planning process for implants in the posterior maxilla but lack the detail required for more comprehensive treatment planning.

To summarise, categorisation of the alveolar ridge using general implant planning classifications helps to guide treatment planning decisions, such as implant diameter, number, position and angulation. Furthermore, they help establish whether adjunctive ridge augmentation is required to optimise implant position and long-term prosthodontic outcomes. However, as these classifications are based on clinical examination alone, posterior maxillary anatomical changes, such as sinus pneumatisation, cannot be evaluated. Therefore, they are not ideal to use for planning of implants in the posterior maxilla. Nonetheless, they are useful for initial clinical assessment and begin the process of determining the surgical and prosthodontic complexity in posterior maxillary edentulous sites.

### Implant planning in the posterior maxilla

The Jensen, Misch (1999) and Juodzbalys and Kubilius classifications listed in Table 3 are specifically designed for implant planning in the posterior maxilla.^38^^,^^39^^,^^40^ Each requires radiographic as well as clinical assessment of alveolar bone, thus enabling planning of implants in the maxillary sinus region. Each categorises radiographic vertical bone height (floor of the sinus to height of the alveolar ridge) using measurements in millimetres. The ideal bone height for implant placement is based on the provision of a ≥10 mm implant, either with or without the need of a sinus augmentation procedure. Each classification describes treatment recommendations for each ridge severity category, including the timing of implant placement (immediate or delayed) and method of sinus augmentation technique (transcrestal or lateral window). The Misch (1999) classification recommends healing periods for graft consolidation and implant osseointegration depending on the augmentation technique or implant timing protocol used.^39^ The Juodzbalys and Kubilius classification includes assessment of the edentulous ridge length and width and alveolar ridge vertical position (defined as distance between the alveolar ridge height and the cemento-enamel junction), which helps determine the surgical and prosthodontic complexity of the case.^40^ However, no further details regarding prosthodontic assessment and treatment planning are provided.

To summarise, the classifications for implant planning in the posterior maxilla enable objective assessment of ridge severity and pre-operative case complexity. Furthermore, they facilitate detailed planning of implant treatment (implant length, position and timing of placement) and sinus augmentation techniques. However, each tends to focus on surgically led planning, with minimal details regarding prosthodontic planning provided.

### Bone quality

Although most implant planning classifications focus on alveolar bone volume to establish case complexity, to comprehensively plan the surgical and prosthodontic needs of implant treatment in the posterior maxilla, bone quality should also be considered. As described in the first article of this series,^41^the bone of the posterior maxilla tends to be of a poorer quality than elsewhere in the jaws. Furthermore, augmented bone in the maxillary sinus is of poorer quality than native bone, particularly in the first six months of graft consolidation.^42^ Bone of lower quality has lower implant-bone contact and lower strength; therefore, there is a higher risk of poor primary stability in the short-term and a higher risk of bone over-loading in the long-term.

The two most commonly referred to classifications relating to bone quality for implant placement are Leckholm and Zarb, and Misch (2021).^30^^,^^42^ These classifications are described in detail in Table 4. Both categorise bone quality in respect to the amount of cortical and cancellous bone present and describe the typical intra-oral location. It is important to note that both classifications identify that the posterior maxilla has a higher amount of sparse trabecular bone and thin cortical bone, which reduces the likelihood of high primary implant stability. The Leckholm and Zarb classification is purely descriptive of the different types of bone found in the mandible and maxilla, and it is not possible to apply this classification using clinical findings. However, the Misch (2021) classification includes a clinical tactile analogue, comparing the tactile feedback during implant osteotomy preparation to the equivalent densities of alternative materials.^42^ It also proposes the likely HUs for each bone quality category, therefore allowing clinical application of this classification. Furthermore, the Misch (2021) classification includes a category for grafted bone and describes this as being the lowest bone quality. These classifications help the clinician consider bone quality as part of planning for implant treatment in the posterior maxilla. They identify the posterior maxilla as being a higher risk zone for implants due to the reduced bone quality, with grafted bone being the poorest bone quality. When considering implants in the posterior maxilla, this highlights the increased complexity of achieving good primary stability and long-term physiologic bone loading in this region. Surgical protocols, implant timing, implant loading protocols and prosthesis design should accommodate for the poorer bone quality, particularly in augmented sinuses.

## Prosthodontic planning classifications

A small number of the already described classifications include elements of prosthodontic assessment to aid pre-operative treatment planning; namely, the ITI SAC, ABC risk score, Misch and Judy, and Juodzbalys and Kubilius classifications.^34^^,^^36^^,^^37^^,^^40^

The ITI SAC and ABC risk score classifications cover a broad scope of factors to aid prosthodontic assessment. The ITI SAC includes: patient expectations; oral hygiene and compliance; craniofacial/skeletal growth; access; number of implants to be placed; prosthesis design (fixed or removable); lip line; biotype; shape of crown; restorative status of neighbouring teeth; tissue contour and volume; inter-arch distance; mesio-distal space; loading protocol; bruxism; use of a provisional restoration; and the retention of the prosthesis (cement- or screw-retained) for the prosthodontic complexity assessment.^36^ The prosthodontic factors assessed in the ABC risk score classification include: biomechanics (no biomechanical problems, implant-tooth connection, unfavourable load distribution, inadequate implant diameter); aesthetics (healthy adjacent tooth, pontics, implant malposition, insufficient space); fixed restoration for full arch (number and distribution of implants, tissue support); and complexity exceeding patient's capability (handling and cleanability).^37^ Both the ITI SAC and ABC risk score classifications are helpful to assess the general prosthodontic complexity for implant planning. However, there is limited focus on the unique factors affecting implant-retained prostheses in the posterior maxilla.

A limited number of prosthodontic factors are included in the Misch and Judy and Juodzbalys and Kubilius classifications.^34^^,^^40^ These include implant angulation, crown height and alveolar ridge vertical position (alveolar ridge to cementoenamel junction). It is encouraging that aspects of prosthodontic complexity are considered in relation to the surgical aspect of treatment; however, the treatment guidance is surgically driven, with no further focus on prosthodontic planning or delivery.

Currently, no classifications consider the aspects of clinical assessment required for restoratively driven planning of implant-retained prostheses in the posterior maxilla. Furthermore, the clinical guidance in each has a surgical rather than a prosthodontic focus for overcoming the unique clinical challenges associated in the posterior maxilla. The second article in this series describes the clinical factors that should be assessed as part of prosthodontic planning, as well as a prosthodontic complexity classification - the Posterior Maxilla Prosthodontic Index (PMPI) - to aid restoratively-driven implant planning.^41^

## Conclusions

Comprehensive understanding of the posterior maxillary anatomy, native bone architecture, and augmented bone architecture is central to providing appropriate prosthodontic and surgical implant treatment. The posterior maxilla presents unique challenges for implant placement due to the anatomical changes in alveolar bone following tooth extraction. The aim of dental implant treatment in the posterior maxilla is to provide a prosthesis that is predictably retained and maintained over time. In order to achieve this, prostheses and implants should be designed to withstand the occlusal forces and prevent bone overload. Numerous classifications are available to help plan the details of dental implant treatment in the posterior maxilla, ranging from simple clinical classifications which assess alveolar ridge shape and volume, to more complex classifications assessing the radiographic extent of the posterior ridge. Furthermore, some classifications include treatment suggestions to help identify the correct treatment in certain clinical situations. However, all current implant planning classifications which can be applied to the posterior maxilla focus on the surgical aspect assessment, planning and treatment, and do not focus on restoratively driven planning.

To aid assessment of the prosthodontic complexity of restoring implants in the posterior maxilla, the second article in this series proposes a prosthodontic complexity classification: the PMPI.^41^ The index focuses on the pre-operative assessment of the horizontal and vertical prosthetic envelope to encourage restoratively led planning for implants placed in the posterior maxilla.

### Supplementary Information

Supplementary Table (PDF 331KB)
