# Correction to: Restoratively driven planning for implants in the posterior maxilla - Part 1: alveolar bone healing, bone assessment and clinical classifications

**DOI:** 10.1038/s41415-023-6502-5

**Published:** 2023-11-10

Author's correction note:

Clinical article *Br Dent J* 2023; **235:** 585-592.

When initially published, there were errors in the author affiliations. These should have read:

Elizabeth M. King*^1^ and Jonathon Schofield^2^

^1^Consultant Senior Lecturer in Restorative Dentistry, University of Bristol, Bristol Dental School, UK; ^2^Senior Clinical Lecturer, University of Bristol, Bristol Dental School, UK.

The authors apologise for any inconvenience caused.

