# Supplementary material for: Restoratively driven planning for implants in the posterior maxilla - Part 1: alveolar bone healing, bone assessment and clinical classifications
Source: Br Dent J. 2023 Oct 27;235(8):585–92. doi: 10.1038/s41415-023-6391-7 (PMC10611561; doi:10.1038/s41415-023-6391-7)
Supplement: Supplementary file 1 — Supplementary Table (PDF 331KB) [file 41415_2023_6391_MOESM1_ESM.pdf]

Supplementary Table 1 General implant planning classifications which can be applied to planning implants in the posterior maxilla. This table contains only relevant elements of each classification. Please see the original publications for the full classification details

Authors	Description	Factors assessed:<br>- Alveolar ridge<br>- Implant planning<br>- Ridge augmentation<br>- Sinus augmentation<br>- Prosthetic planning	Classification categories	Case complexity / severity	Treatment recommendations
Cawood & Howell[27]	Classifies edentulous maxillae and mandibles according to the degree of alveolar resorption	- Alveolar ridge	I) dentate<br>II) immediately post extraction<br>III) well rounded ridge form, adequate height and width<br>IV) knife-edge ridge form, adequate in height and inadequate in width<br>V) flat ridge form, inadequate in height and width<br>VI) depressed ridge form, with some basal bone loss evident	Severity identified by ridge shape only	No
Leckholm & Zarb[28]	Classifies edentulous maxillae and mandibles according to the degree of alveolar resorption and histological appearance	- Alveolar ridge	A) intact ridge<br>B) moderate resorption<br>C) advanced resorption extending to basal bone<br>D) initial resorption of basal bone<br>E) extreme resorption of basal bone	Severity identified by ridge shape and/or bony histology	No
Siebert[30]	Classifies pattern of alveolar bone loss in relation to the need to surgically reconstruct with onlay grafts for satisfactory ridge:pointic relationship.	- Alveolar ridge	Class I – buccolingual loss of tissue with normal apicocoronal ridge height<br>Class II – apicocoronal loss of tissues with notmal buccolingual ridge width<br>Class III – combination-type defects (loss of both height and width)	No severity scale	No
Modified Siebert[31]	Classifies pattern and severity of alveolar bone loss in relation to the need to surgically reconstruct with bone augmentation for	- Alveolar ridge	A – apicocoronal loss of tissue<br>B – Buccolingual loss of tissue<br>C – combination	Mild (< 3mm resorption);<br>Moderate (3-6 mm resorption);<br>Severe (> 6 mm resorption)	No

	satisfactory ridge:pointic relationship.							
Misch & Judy[32]	Alveolar ridge classification based on Kennedy classification:<br>Class I: Bilateral free end saddles<br>Class 2: Unilateral free end saddle<br>Class 3: Posterior bounded saddle<br>Class 4: Anterior bounded saddle	- Alveolar ridge<br>- Implant planning<br>- Ridge augmentation<br>- Prosthetic planning	***Group***	***Ridge width (mm)***	***Ridge height (mm)***	***Crown height (mm)***	***Implant angulation (degrees)***	***Treatment recommendations***
			A – Abundant	> 6	> 12	< 15	< 25	Regular or wide diameter implants; Osteoplasty
			B – Barely sufficient	2.5-6	> 12	< 15	< 25	Narrow diameter implants; osteoplasty
			C – Compromised	< 2.5	< 12	> 15	> 30	Short implants; bone augmentation
			D – Deficient	Severe atrophy including basal bone	Severe atrophy including basal bone	> 20	> 30	Substantial bone augmentation (extra-oral site)
HVC ridge deficiency[33]	Classifies pattern and severity of alveolar bone resorption with associated treatment recommendations	- Alveolar ridge<br>- Ridge augmentation	H – Horizontal	Small ≤ 3 mm resorption				***H-Small***: Ridge expansion; inlay; onlay; GBR*<br>***H-Medium***: Inlay; onlay; GBR*<br>***H-Large***: Inlay; onlay; GBR*
			V – Vertical	Medium 4-6 mm resorption				***V-Small***: Ortho extrusion; GBR*<br>***V-Medium***: Ortho extrusion; GBR; onlay; distraction osteogenesis<br>***V-Large***: GBR*; onlay; Distraction osteogenesis
			C – Combination	Large ≥ 7 mm resorption				***C-Small***: Inlay; onlay; GBR*<br>***C-Medium***: Inlay; onlay; distraction osteogenesis<br>***C-Large***: Exraoral block graft (difficult, multiple procedures needed)
SAC Assessment Tool[34]	An online tool which assesses different clinical factors (surgical and prosthodontic) to allocate cases into three different complexity groups.	- Alveolar ridge<br>- Implant planning<br>- Ridge augmentation<br>- Sinus augmentation<br>- Prosthetic planning	Simple	Horizontally and vertically sufficient				No augmentation required
			Advanced	Deficient horizontally allowing simultaneous augmentation				Simultaneous horizontal bone augmentation

	The aim is to provide a pre-operative assessment of the risks associated with the implant case. All individual pre-operative factors are input and evaluated to generate an overall complexity scoring.		Complex	Deficient horizontally requiring prior grafting		Staged horizontal bone augmentation
				Deficient vertically or deficient vertically AND horizontally		- Staged vertical and/or horizontal bone augmentation<br>- Sinus floor graft in posterior maxillary sites
ABC Risk Score[35]	Different clinical factors are assessed and a scoring system is used to allocate cases into a risk group which defines the complexity of treatment. This helps identify whether implant treatment is low, medium or high risk or not recommended.	- Alveolar ridge<br>- Implant planning<br>- Ridge augmentations<br>- Sinus augmentation<br>- Prosthetic planning	**Risk score**	**Ridge defect**	**Augmentation**	No
			Always – lowest assessed risk (green)	- No ridge defect or/<br>- Horizontal deficiency with local bony walls<br>- Vertical deficiency with local bony walls	- No augmentation necessary<br>- Horizontal with bony support<br>- Sinus floor elevation, septae	
			Between – medium risk (yellow)	- Horizontal deficiency with no bony walls<br>- Vertical deficiency with no bony walls	- Horizontal without bony support<br>- Sinus floor elevation, internal (>2 mm)<br>- Vertical	
			Complex – increased risk (orange)	NA	NA	

*GBR = Guided bone regeneration
